# The recording of adverse events from psychological treatments in clinical trials: evidence from a review of NIHR-funded trials

**DOI:** 10.1186/1745-6215-15-335

**Published:** 2014-08-27

**Authors:** Conor Duggan, Glenys Parry, Mary McMurran, Kate Davidson, Jane Dennis

**Affiliations:** Institute of Mental Health, University of Nottingham Innovation Park, Triumph Road, Nottingham, NG7 2TU UK; Head of Research & Development, Partnerships in Care, 2 Imperial Place, Maxwell Road, Borehamwood Herts, WD6 & JN UK; Centre for Psychological Services Research, University of Sheffield, School of Health and Related Research, Regent Court, 30 Regent Street, Sheffield, S1 4DA UK; The Institute of Mental Health, University of Nottingham Innovation Park, Triumph Road, Nottingham, NG7 2TU UK; Institute of Health and Wellbeing, College of Medical, Veterinary and Life Sciences, University of Glasgow, Gartnavel Royal Hospital, 1st Floor, Administration Building, 1055 Great Western Road, Glasgow, G12 0XH Scotland; Campbell Collaboration, Social Well Fare Group, 86 Cranbrook Road, Bristol, BS6 7DB UK

**Keywords:** Harm, Adverse events, Psychological interventions, Clinical trials

## Abstract

**Background:**

There is a concern in the literature that harm from interventions is insufficiently documented in clinical trials in general, and in those assessing psychological treatments in particular. A recent decision by a trial steering committee to stop recruitment into a randomized controlled trial (RCT) of a psychological intervention for personality disorder led to an investigation of the recording of harm in trials funded by the National Institute for Health Research (NIHR).

**Methods:**

The protocols and final reports of all 82 NIHR trials funded between 1995 and 2013 were examined for the reporting of adverse events. These were subdivided by category of intervention.

**Results:**

None of the psychological intervention trials mentioned the occurrence of an adverse event in their final report. Trials of drug treatments were more likely to mention adverse events in their protocols compared with those using psychological treatments. When adverse events were mentioned, the protocols of psychological interventions relied heavily on severe adverse events guidelines from the National Research Ethics Service (NRES), which were developed for drug rather than psychological interventions and so may not be appropriate for the latter.

**Conclusions:**

This survey supported the belief that the reporting of adverse events in psychological treatments is weak and the criteria used may not be appropriate. Recommendations are made as to how current practice might be improved.

## Background

During 2012, the Data Monitoring Committee (DMC) of a publicly funded trial of a psychoeducation with problem-solving (PEPS) intervention for community-dwelling adults with personality disorder [1] noted higher rates of adverse events (AEs), specifically mental health-related adverse events (overdose, self-harm and suicide attempts), among those in the active arm of the intervention. The DMC, who examined unblinded data, alerted the Trial Steering Committee (TSC), whose role is independent trial oversight. The TSC were unable to rule out the possibility that the intervention was instrumental in the excess of AEs and so the TSC issued an instruction to stop recruitment into the trial. To our knowledge, this was the first time recruitment to a UK trial of a psychological therapy had been stopped on the grounds of adverse events.

While there is recognition that harm might arise from psychological interventions in the training of those who provide psychological therapies [2] and in the theoretical psychotherapy literature [3], many believe that this is neglected in both research and the actual practice of psychotherapy. Several commentators have criticized, for instance, the assumption that as psychotherapy is only talking, the focus has been on its benefits rather than on the harm that it might cause [4, 5]. Parry [6] has argued that the increasing recognition that psychological interventions can be effective might also lead to a parallel recognition of the harm that they may cause. Others have contrasted the scrutiny which is applied to medication, where adverse monitoring is mandatory, with evaluation of psychotherapy [5]. Although there is growing interest in this issue [7, 8], it nonetheless does not appear to affect the recording of harm (or lack thereof) in clinical trials that assess psychological interventions.

This review examines the recording of AEs in trials of psychological treatments in a particular funding stream, namely the Health Technology Assessment (HTA) program of the UK’s National Institute of Health Research (NIHR), and makes some recommendations to improve practice in the recording of such events in future trials.

### Adverse events and harm

**Figure 1:**
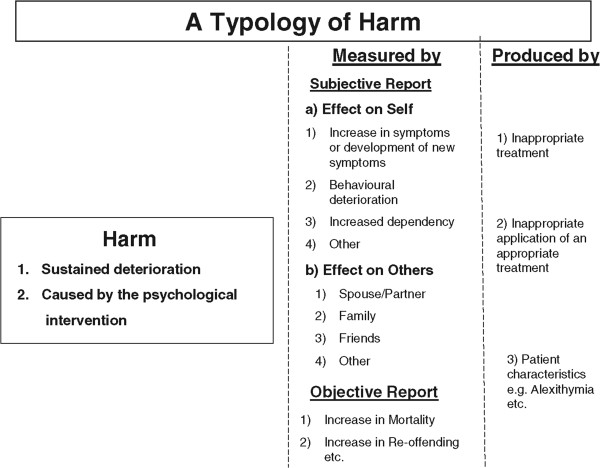
**A definition of harm from a psychological intervention together with its measurement and etiology.**

We define ‘harm’ (in which an adverse event is a particular instance) as a sustained deterioration that is caused directly by the psychological intervention (Figure 1). The fact that the deterioration has to be sustained addresses the concern that some temporary discomfort is often a necessary part of the process of psychological change [9].

It is also important to establish a causal connection between the intervention and the subsequent deterioration, as the latter may be due to other causes (for example, a part of the natural history of the condition or a response to troubling life events). For instance, when a depressed suicidal patient commits suicide despite being in treatment, it would be wrong to automatically assume that the treatment was responsible. While the distinction between harm being caused by the intervention or being part of the natural course of the disorder or due to life events is probably impossible in an individual case, the design of a randomized trial permits consideration of this possibility when there is a differential effect between the trial and the control condition. Lilienfeld [7] makes use of the importance of trial evidence by making it a further criterion in establishing harm arising from a psychological intervention, namely that the harm has been replicated in a number of good quality clinical trials using the intervention, but he recognizes the ethical difficulty in setting up a clinical trial to replicate an iatrogenic effect. Consequently, we have not included that in our definition of harm.

The second consideration is how harm is measured (illustrated in the second column in Figure 1). There are two sources of evidence: subjective and objective, with both subdivided into that which is reported by self and others. The objective measures could comprise both patient-reported outcomes using psychometrically valid and reliable instruments and clinician-rated objective measures. These subdivisions illustrate the complexity of the area if the assessment of harm is to capture the multiple domains of an individual’s functioning and where a perceived benefit to one party may not always shared by others (such as in couples therapy). Similarly, self-reported improvement and satisfaction with the treatment does not always accord with improvement assessed by pre-post differences in psychometric outcome scores, or when rated objectively by outside assessors. We make a distinction between perceived harm and that defined through objective measurement.

Third, there is a process question - how has this harm occurred (third column in Figure 1)? We divide this into a simple trichotomy of being caused by: (a) the treatment, (b) the application of the treatment, or (c) patient-related factors. In (a), harm may have been caused directly by using an inappropriate treatment which results in clinical deterioration, or when an ineffective treatment for an individual’s condition is chosen despite a more effective option being available (the potential to cause harm by withholding or delaying a beneficial treatment). In (b), harm may arise when an otherwise effective intervention has been inappropriately applied. This is especially important for psychological treatments where the competence and natural attributes of the therapist assume greater importance compared to those of a practitioner prescribing a drug [10]. Finally (c), where attributes of the patient may render an appropriate treatment applied by a competent therapist ineffective. For instance, it is recognized that while relaxation treatment may be helpful to some who suffer from panic attacks, for others, its focus on interoceptive stimuli can have the effect of precipitating attacks [11]. Thus, an intervention may be effective overall but be harmful to a minority.

We illustrate the utility of this harm matrix by applying it to the findings from the Cambridge Somerville Youth Trial, in which matched delinquent youths were subsequently randomized either to the active treatment condition (a supportive counselling intervention that continued for five years) or to whatever was available from current services [12]. When those in the trial were followed up 30 years after its completion, it was found that while there was no benefit from the active treatment in 59% of the matched pairs, in the 41% where there were differences, those with the worse outcomes across a range of measures had been in the active treatment condition [13]. These included increases in mortality, rates of mental disorder and alcoholism, re-offending and lower levels of occupational attainment. The range of outcomes and the long time frame strongly indicate that those in the active arm of the trial had suffered sustained harm. This was in spite of the fact that both the children and their parents in the active arm had expressed their appreciation of the help that had been provided by their counsellors. It was also argued that the deterioration had been caused by the intervention as those that fared worst had received the most help (there was a dose response relationship), and were children of the most compliant parents [13]. The trial was also important in that its investigators sought to provide a mechanism to explain this unexpected finding. Although various possibilities were considered, such as the raising of expectations that were unsustained once the intervention was withdrawn, and ‘deviancy training’ where the more delinquent youth had a detrimental influence on their more vulnerable peers while they were together at summer camp [14], the cause of this adverse outcome has proved to be elusive [15]. This highlights one of the major problems in this area, namely, that it is difficult to be sure why harm has occurred, even when harm has been established.

### Adverse events in clinical trials

We define an adverse event as harm occurring within a trial where there is at least an assumption of causality. This may have been anticipated (and therefore identified within the protocol of the trial) or unanticipated - the latter being some untoward occurrence that requires attention even though it was not noted in the original protocol. Often, interventions are offered to vulnerable individuals and one ought not to be surprised if untoward outcomes occur. Here, a randomized design assists in clarifying the issue as the crucial distinction is that the differential rate of untoward outcomes can be assessed between the active and control condition.

The difficulties in defining an adverse event (particularly in establishing causality) are acknowledged. For instance, in an update of the CONSORT statement to improve the reporting of harm, Ioannidis *et al*. [16] write: ‘In a typical randomized trial, it is difficult to know whether an observed event is partially or entirely due to the intervention or whether it is totally unrelated to the intervention. The purpose of the trial is to collect and appropriately report good and bad events and outcomes so that they may be compared across treatment groups. In this respect, the term "adverse events" is probably better to describe harmful events that occur during a trial’ (p. 782). Serious adverse events are defined as ‘…reactions which, in their most severe forms, threaten life or function. Such reactions should be promptly reported to regulators’ ( [16], p. 782).

This focus on the safety of the intervention requires that any specified adverse events, along with any serious unexpected untoward events, must be recorded and reported by the Chief Investigator to a range of bodies - the Research Ethics Committee, the TSC, and the DMC. Of these, only the DMC has access to unblinded data and is required to provide an independent review and recommendations in the light of potential treatment adverse effects. The DMC must then make a decision on whether to inform the TSC. It is the TSC that has the responsibility to recommend discontinuation of the study if significant ethical or safety concerns arise or if there is unequivocal statistical evidence of benefit prior to the completion of the study. However, these decisions are rarely straightforward, and there is a different threshold for stopping a trial in the case of potential harm than in the case of benefit [17]. In the PEPS trial, the TSC recommended that recruitment and treatment of participants should cease due to the excess number of adverse events relating to mental health in the active treatment arm of the trial, although the trial should continue to collect follow-up data on all of those who had already completed treatment.

One other contentious issue is whether non-completion of treatment (also known as leaving early, premature termination or dropout) ought to be considered an adverse event. The reasons for dropping out are heterogeneous and some reviews have considered drop out as an adverse event [18].

### The reporting of adverse events in clinical trials of psychological interventions

The recognition that the reporting of harms in randomized controlled trials is unsatisfactory led to the CONSORT group suggesting 10 new recommendations in the reporting of harms [16]. These included: more comprehensive definitions of harm and how this will be monitored to be stated in the Method; reporting of any discontinuation or reduction of dosage during the study; if no adverse events are recorded, then this should be explicitly acknowledged within the Results; and, finally, a balanced appraisal of the benefits and risks of the intervention to be provided in the Discussion.

Despite these injunctions, there remain inadequacies of adverse event reporting in non-pharmacological interventions in general and particularly in mental health trials. Ethgen *et al*. [19] showed that in the reporting of harms, withdrawals due to adverse events and severity were more frequent in pharmacological compared with non-pharmacological treatments in rheumatoid arthritis and osteoarthritis. In mental health, the reporting of harm in drug trials was adequate in only 21.4% of the trials reviewed but none of the non-drug trials had adequate reporting of clinical adverse events [20].

We examined the reporting of adverse events in recent trials of psychological treatments for those with personality disorder (a similar patient population to PEPS). Of 39 trials for antisocial (ASPD) and borderline personality disorder (BPD), Gibbon *et al*. [21] found only one out of 11 studies mentioned adverse events for ASPD, and Stoffers *et al*. [22] found none for BPD. Jonsson *et al*. [23], in a recent review, reported that only 21% of randomized controlled trials of psychological intervention had information suggesting that harm was monitored.

We investigated this issue in trials funded by the National Institute of Health Research (NIHR), the main public funder of psychological therapy trials in the UK. Our objectives were: (a) to discover whether PEPS is the first such trial to be discontinued for this reason, (b) to report the extent to which adverse events are specified in psychological therapy trial protocols, and (c) reported in trial reports.

## Method

On request, NETSCC (NIHR Evaluation, Trials and Studies Coordinating Centre) sent the investigators a list of all studies funded between August 1995 and October 2013. There were 102 studies: 85 funded by the HTA, 10 by Public Health Research (PHR), 5 by Efficiency and Mechanism Evaluation (EME) and 2 by the Public Services and Delivery Research (PS & DR) programs. Studies that involved a clinical evaluation of any kind were included. This comprised 91 studies, which included all 85 which were funded through the HTA program, 5 by the EME program and 1 by the HS & DR program. These comprised pharmacological, psychological and other interventions for mental health (71%) and other physical conditions (such as diabetes and obesity). Of these, nine were excluded, either because they were not trials (for example epidemiological surveys) or because there was insufficient information available for their inclusion.

**Figure 2:**
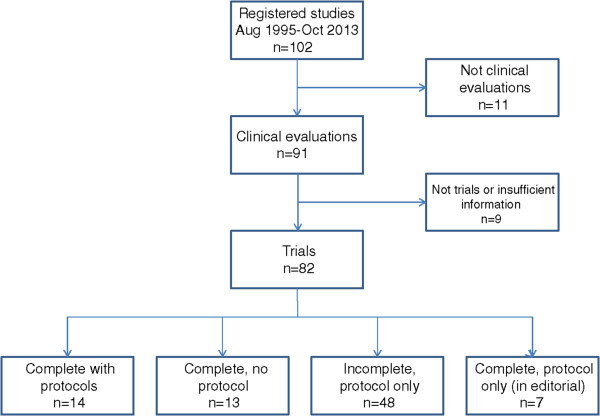
**Flow diagram of included and excluded trials from the NIHR database.**

We divided these 82 trials into four groups: (a) completed trials with a final report and protocol, (b) completed trials with a final report but without a protocol, (c) incomplete trials which had been recently funded and where only the protocols were available, and (d) incomplete trial reports in the editorial stage (again, where only the protocols were available as the final report is awaited, see Figure 2.)

The trials were subdivided into those which were (a) psychological only, (b) drug only, (c) a combination of psychological and drug, and (d) other (such as occupational therapy, physiotherapy or case management).

These 82 reports were searched manually by the first author and any information on adverse events from either the protocol and/or final reports (where available) was extracted and summarized.

## Results

In the 82 trials, 28 trials had been published with a final report; 14 of these had protocols available but another 14 did not. For the latter we relied on the published report only. Of the remaining 54 trials, 47 were still in progress and another 7 were in the process of being published so that for these only the protocols were available for examination. A total of 44 of the interventions were psychological, 14 involved drugs, 5 were a combination of drugs and a psychological intervention and 19 were other (see Table 1).

**Table 1:** **Recording of adverse events (AEs) from 82 NIHR-funded trials subdivided by types of intervention**

		Number of Protocols	Psychological Treatments	Drug Treatments	Combined	Other
Completed studies with protocols	Number of protocols	14	7	3	2	2
	Number with AEs	5	2 (28%)	1 (33%)	1 (50%)	1 (50%)
	AEs in final report	3	0	1	1	1
Completed studies without protocols	Number of final reports	13	7	2	1	3
	Number with AEs	1	0	0	0	1 (33%)
Incomplete studies (protocols only)	Number of protocols	48	28	8	2	10
	Number with AEs	31	17 (61%)	8 (100%)	2 (100%)	5 (50%)
Incomplete studies (in editorial, protocols only)	Number of protocols	7	2	1	0	4
	Number with AEs	4 (50%)	0	1 (100%)	0	3 (75%)

Results from survey of 82 NIHR-funded trials.

### Completed studies

We found no evidence of a trial having been discontinued because of adverse event reports prior to PEPS. Specifically, none of the trials using a psychological treatment were stopped because of an adverse event differential. In none of the 14 completed studies using a psychological intervention was there a mention of an adverse event. Conversely, there was some reporting of adverse events in the final reports of each of the other categories.

### Incomplete studies

In the incomplete trials and those at the editorial stage, we found that psychological interventions (55% of the total) mentioned adverse events in 17 of the 30 trial protocols (56%). In contrast, drug interventions (together with drug and psychological interventions combined) mentioned adverse events in all of the 9 protocols examined. We noted, however, that in the more recently funded psychological studies that adverse events were more likely to be mentioned in their protocols.

In summary, none of the psychological interventions mentioned adverse events in their final reports and were less likely to list adverse events in their protocols compared with drug trials but this difference is changing with the more recently funded psychological trials at least mentioning adverse events in their protocol.

Looking specifically at how adverse events were recorded in the protocols of these trials of psychological interventions, there appeared to be two approaches. The first was for these not to be considered at all, or for the likelihood of their occurrence to be dismissed. For instance, one finds such comments as ‘There is no evidence that CBT is harmful. IAPT therapists are experienced in conducting risk assessment’ and ‘No adverse events or serious adverse events will be recorded or reported in this study’. Alternatively, when adverse events were reported there was a reliance on the definitions of severe adverse events from the safety reporting guidelines from the National Research Ethics Service (NRES). Here, a severe adverse event is one which: (a) results in death, (b) is life threatening, (c) requires hospitalization or prolongation of existing hospitalization, (d) results in persistent or significant disability, (e) consists of congenital abnormality or birth defect or (f) is otherwise considered medically significant by the investigator. When adverse events are listed in the protocols of psychological interventions, one can see the influence of NRES, with death occurring in 65%, hospitalization in 56%, persistent disability in 43%, and congenital abnormality or birth defect in 39%. Other events that one might consider relevant for psychological therapies (such as self-harm, symptom change, distress or attendance at Accident & Emergency departments) were mentioned in only a few trials.

## Discussion

Randomized controlled trials provide the best opportunity to examine the recording of adverse events as data are more likely to be systematically recorded compared with routine clinical practice, and their design allows one to draw causal inferences between an intervention and any harmful outcome [8]. They therefore offer an opportunity, not only to identify and thereby avoid using interventions that might be harmful, but also to provide information about possible mechanisms whereby psychological interventions can effect change.

There is substantial evidence that some patients may be harmed by psychological therapy, although the prevalence and causes of harm have been inadequately researched ( [24], p. 206). Indications of potential harm are adverse events, deterioration in mental state and the patient’s perceptions of having been adversely affected. This study focused on the first of these indicators, adverse events, which one would expect to be routinely recorded and reported in well-conducted randomized controlled trials. However, our examination of the literature suggests that unfortunately such events are not being adequately reported. The failure to specify, record and report adverse events during psychological therapy was confirmed by our examination of 91 NIHR-funded trials.

This absence of reporting could mean that no adverse event occurred (a zero event), that it occurred but it was either not recognized or recorded, and finally, that it was recorded but not reported. Current practice, regrettably, does not allow one to distinguish between these alternatives.

Of the 37 psychological trial protocols examined, 17 (46%) did mention adverse events, but used the definitions of adverse events suggested by the NRES, for example death, hospitalization or persistent disability. There was little evidence of thought being given to which adverse event might be likely to arise from a specific intervention in a particular population. For example, people with borderline personality disorders may be at increased risk of negative outcomes from psychological therapy [25] and whilst some hospital attendance for minor self-harm is characteristic of this population, life-threatening self-harm and relationship breakdown could be considered adverse events.

An example of how defining therapy-specific adverse events could be achieved is provided by Horigian *et al*. [26] who produced some general principles and gave some examples to help illustrate their definition. In their trial of family therapy for drug abusing adolescents, for instance, they did not consider an increase in drug use to be an adverse event, arguing that drug use among adolescents is so erratic that any increased usage would be difficult to measure. Hence, it was only when such increased drug use resulted in hospitalization that it was categorized as a severe adverse event. In addition, they recommended systematic monitoring so that general and specific questions that target the relevant areas are asked throughout the study at specified intervals. To achieve comprehensive coverage of harm, experts in the intervention, ethicists and medical safety officers were all involved in adverse event definition and monitoring during the development of the protocol for the trial.

Horigian *et al*. [26] also highlight a further issue which requires attention: only some adverse events are spontaneously reported, and accurate assessment requires systematic monitoring, with the use of structured interviews and records from other agencies. Horigian *et al*. [26] found that only 30% of adverse incidents were spontaneously reported to research assistants, but this increased to 70% when structured interviews were used. This is consistent with findings that psychotherapy patients are likely to hide negative material in particular, and that even experienced therapists are rarely able to detect this [27].

There was evidence of improvements over time, with more recent trials more likely to specify adverse events in protocols. Over the time period covered by this study, there have been a number of developments in accepted good practice in trial design and reporting, which take time to filter through to protocols and even longer to influence final reports. For example, the 2004 revision of the CONSORT recommendations on harm monitoring seems to have had little impact in our study sample. The anticipated guidance on the reporting of complex randomized controlled trials within CONSORT-SPI [28], which we understand will include advice on the recording of adverse events, is to be especially welcomed and research funding agencies and trial investigators should consider how to expedite its adoption.

The revised EU clinical trials directive^a^ will govern pharmacological trials, but is likely to influence the conduct of trials of psychological interventions in the UK and continental Europe. The revisions aim to introduce a simpler, risk-adjusted authorization procedure for trials of medicines and medicinal products and simpler processes for reporting suspected serious adverse reactions. Whilst it is unlikely that psychological interventions would be included in such regulations in the foreseeable future, bringing such reporting to an equivalent standard would address many of the shortcomings we identified in trial protocols and reports.

Before presenting recommendations, we need to consider the limitations of this study. First, the small numbers of trials in each group make it difficult to be confident that the differences found between psychological and pharmacological trials, or between earlier and later protocols, are robust. The number of earlier trials with no accessible protocol was a result of the funding agency not insisting that protocols were produced. This omission has now been corrected so that all recent trials have a detailed protocol. Despite these limitations, it is clear that there is considerable room for improvement in specifying, recording and reporting adverse events in NIHR-funded clinical trials of psychological interventions. We therefore discuss possible ways to address these shortcomings.

Firstly, trialists could report on the full range of scores on the principal dependent variables distributed by quartile or, for dichotomous variables, the number needed to harm rather than the group mean, as it is likely that the active treatment will increase the variance compared to no treatment control [7]. Alternatively, or in addition, investigators could report the proportion of patients who deteriorate on clinical measures in both the experimental and control groups. At an individual level, visual inspection of the outliers in the fourth quartile may identify those at risk of being harmed by the intervention [8]. This recognition of an adverse effect of treatment at an individual level is important as it allows an identification of clinical deterioration in certain instances even when the intervention overall has been successful. Secondly, papers should explain how adverse events were defined and recorded, and should report AEs for treatment and control groups. If AEs have not been detected then this should be stated explicitly in the results. Investigators should describe the method they used to determine whether or not adverse events are related to the treatment intervention. Thirdly, investigators should not rely on generic checklists but try to identify, by consensus, adverse effects that could plausibly be related to the effect of the intervention on the population being studied. These include being informed by previous research into the prevalence of the disorder, its natural progression and symptomatology.

Fourthly, active and systematic methods should be used to capture as complete a dataset as possible when recording adverse events, in both treatment and control groups, including structured interviews and use of service utilization databases. Fifthly, whilst we have argued that current practice suffers from insufficient recording of adverse events when psychological interventions are evaluated in clinical trials, one must be careful not to err in the opposite direction leading to the over-recording of information. Already, commentators argue that the regulatory framework for conducting large randomized trials is already so burdensome that trials are very difficult and costly to carry out [29, 30]. Given the multifaceted consequences of psychological interventions described in Figure 1, there is a temptation to develop a long list of potential adverse effects that would be difficult to monitor and interpret. This needs to be resisted. What is needed is a streamlined yet flexible list of events that are meaningfully related to the condition and intervention that are informed by the trialists’ expertise and the patients’ needs. This will require thought and effort from both parties and ought not to be seen as an add-on to other aspects of the design of the investigation.

While clinical trials are difficult to carry out, their design provides the most internally valid estimates of harmful effects [8]. Although there are difficulties in studying rare events (such as harms) in trials with modest sample sizes and other methods such as large N observational studies and intensive process analysis are also required [31], only a properly randomized comparison allows conclusions to be drawn about the relative rates of adverse effects and adverse events between interventions, as well as their relative efficacy. If the reporting of negative outcomes and adverse events in trials were improved, meta-analysis would enable robust conclusions to be drawn about the risks and benefits of psychological therapies

As Dimidjian and Hollon [8] point out, identification of a harmful effect is only the first step; what is then required is an understanding of the mechanism to explain such an effect (such as vicarious learning from a negative role model in group program for delinquents). Such an understanding would not only lead to the avoidance of harmful interventions, but also contribute to identifying which psychological intervention works best for whom.

## Conclusions

This study examined the reporting of adverse events across a range of interventions in NIHR-funded trials between August 1995 and October 2013. We found that those involved in trialing psychological interventions gave less attention to the reporting of adverse events compared with trials of other types of interventions and especially with drug interventions. Although this situation is improving with more recently funded psychological trials mentioning adverse events in their protocol, the types of adverse events described are strongly influenced by categories from the NRES safety reporting standards which have limited applicability for psychological interventions. We conclude with some recommendations for the future practice of recording and reporting adverse events in clinical trials of psychological interventions. In particular, we recommend less reliance on generic checklists, but rather on events that could plausibly be related to the condition being treated, given its natural progression and symptomatology.

## Endnote

^a^Regulation (EU) No 536/2014 of the European Parliament and of the Council of 16 April 2014 on clinical trials on medicinal products for human use, and repealing Directive 2001/20/EC.

## Abbreviations

AE: Adverse event
ASPD: Antisocial personality disorder
BPD: Borderline personality disorder
CONSORT-SPI: Consort extension for social and psychological interventions
DMC: Data Monitoring Committee
EME: Efficiency and Mechanism Evaluation
HTA: Health Technology Assessment
NETSCC: NIHR Evaluation, Trials and Studies Coordinating Centre
NIHR: National Institute of Health Research
NRES: National Research Ethics Service
PEPS: Psychoeducation with problem-solving
PHR: Public Health Research
PS & DR: Public Services an Delivery Research
RCT: Randomized Controlled Trial
TSC: Trial Steering Committee.
